# Amphotericin B Inhibits Enterovirus 71 Replication by Impeding Viral Entry

**DOI:** 10.1038/srep33150

**Published:** 2016-09-09

**Authors:** Fengwen Xu, Xiaoxiao Zhao, Siqi Hu, Jian Li, Lijuan Yin, Shan Mei, Tingting Liu, Ying Wang, Lili Ren, Shan Cen, Zhendong Zhao, Jianwei Wang, Qi Jin, Chen Liang, Bin Ai, Fei Guo

**Affiliations:** 1 MOH Key Laboratory of Systems Biology of Pathogens, Institute of Pathogen Biology, and Center for AIDS Research, Chinese Academy of Medical Sciences & Peking Union Medical College, Beijing, P. R. China; 2 Institute of Medicinal Biotechnology, Chinese Academy of Medical Sciences & Peking Union Medical College, Beijing, P. R. China; 3 Lady Davis Institute, Jewish General Hospital, Montreal, Qc, Canada H3T 1E2; 4 Department of Medical Oncology, Beijing Hospital, Beijing, P. R. China

## Abstract

Enterovirus 71 (EV71) infection causes hand-foot-and-mouth disease that leads to cardiopulmonary complications and death in young children. There is thus an urgent need to find new treatments to control EV71 infection. In this study, we report potent inhibition of EV71 by a polyene antibiotic Amphotericin B. Amphotericin B profoundly diminished the expression of EV71 RNA and viral proteins in the RD cells and the HEK293 cells. As a result, EV71 production was inhibited by Amphotericin B with an EC50 (50% effective concentration) of 1.75 μM in RD cells and 0.32 μM in 293 cells. In addition to EV71, EV68 was also strongly inhibited by Amphotericin B. Results of mechanistic studies revealed that Amphotericin B targeted the early stage of EV71 infection through impairing the attachment and internalization of EV71 by host cells. As an effective anti-fungi drug, Amphotericin B thus holds the promise of formulating a novel therapeutic to treat EV71 infection.

Enterovirus 71 (EV71) is a member of the *Picornaviridae* family. Its main target population are children who, upon infection with EV71, develops rashes, diarrhea and hand-foot-and-mouth disease (HFMD)^1^,^2^,^3^. In severe cases, EV71 infection leads to central nervous system diseases^4^,^5^,^6^. Since the isolation of EV71 in 1969^7^, EV71 infections have caused a series of epidemics in the western Pacific region countries, including China, Japan, Malaysia, and Singapore^8^,^9^,^10^,^11^,^12^.

EV71 has a single-stranded positive-sense RNA genome that encodes a single precursor protein. This precursor protein is cleaved by viral protease into mature structural and non-structural proteins. Several cell surface proteins have been reported to either serve as the receptors of EV71, including human P-selectin glycoprotein ligand-1 (PSGL-1), scavenger receptor B2 (SCARB2) and heparan sulfate^13^,^14^,^15^,^16^, or to promote EV71 entry such as vimentin^17^.

A number of drugs have been reported to inhibit of EV71 infection. For example, the entry or uncoating of EV71 is impaired by Pleconaril, picodavir, and BPROZ-194^18^,^19^,^20^. Rupintrivir inactivates the proteases of human rhinovirus and EV71^21^,^22^,^23^,^24^. The attachment of EV71 to cells is strongly inhibited by glycosaminoglycans^25^. DTriP-22 and aurintricarboxylic acid inhibit viral RNA-dependent RNA polymerase 3D^26^,^27^. The RNA polymerase of EV71 is inhibited by nucleoside analogs such as ribavirin, 2′-C-methylcystidine, and N-6-modified purine^28^,^29^,^30^,^31^. However, none of these drugs have been approved for clinical treatment of EV71 infection. Discovery of new EV71 inhibitors is thus urgently needed. Long term and high cost of antiviral development make the use of drug products in low- and middle-income countries extremely limited. It has therefore been an important alternative strategy to discover new applications for old drugs. In this context, we have tested Amphotericin B as a potential EV71 inhibitor.

Amphotericin B has been used to treat serious systemic fungal infection such as Aspergillosis since the 1950s. Multiple clinical formulations of Amphotericin B are currently used to treat a growing number of fungal infections^32^. Amphotericin B kills fungi and single cell parasites like Leishmania spp by preferentially binding to ergosterol than cholesterol. In addition, Amphotericin B also exhibits antiviral activity against vesicular stomatitis virus (VSV), herpes simplex virus types 1 (HSV-1), Sindbis virus, vaccinia virus and human immunodeficiency virus type 1 (HIV-1)^33^,^34^,^35^,^36^,^37^,^38^,^39^. Recently, it was utilized in combination therapy to treat co-infection by fungi and viruses^40^,^41^. In this study, we have shown that Amphotericin B inhibits EV71 infection by directly blocking the attachment and internalization of EV71 to host cells. These results suggest that Amphotericin B has the potential of becoming a new treatment of EV71 infection.

## Results

### Amphotericin B inhibits EV71 infection

**Figure 1:**
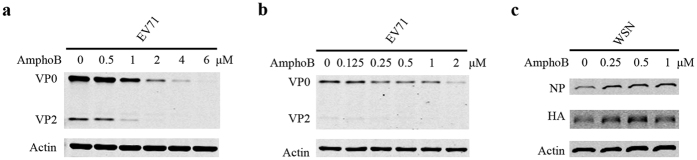
Amphotericin B inhibits EV71 infection. (**a**) RD cells were pretreated with increasing concentrations of Amphotericin B for 2 h, and then exposed to EV71 at an MOI of 4. At 8 h post infection, levels of EV71 proteins in cells were determined by Western blotting. AmphoB: Amphotericin B. (**b**) Similar experiments were performed in 293 cells using EV71 at an MOI of 25. (**c**) A549 cells were pretreated with Amphotericin B at the indicated concentrations for 2 h and then inoculated with WSN33 at an MOI of 0.002. At 16 h post infection, levels of influenza viral proteins NP and HA were determined by Western blotting. The complete blots are presented in Supplementary Fig. 1.

We first measured the effect of Amphotericin B on EV71 (Fuyang) infection of RD cells (Fig. 1a). EV71 infection was monitored by measuring levels of EV71 viral protein in RD cells. The results showed that Amphotericin B profoundly diminished the expression of EV71 proteins VP0 and VP2 as increasing concentrations of Amphotericin B were used. Similar inhibition was observed in EV71 infection of 293 cells (Fig. 1b). In contrast, Amphotericin B increased influenza A virus infection (Fig. 1c), which is consistent with what has been previously reported^42^.

**Figure 2:**
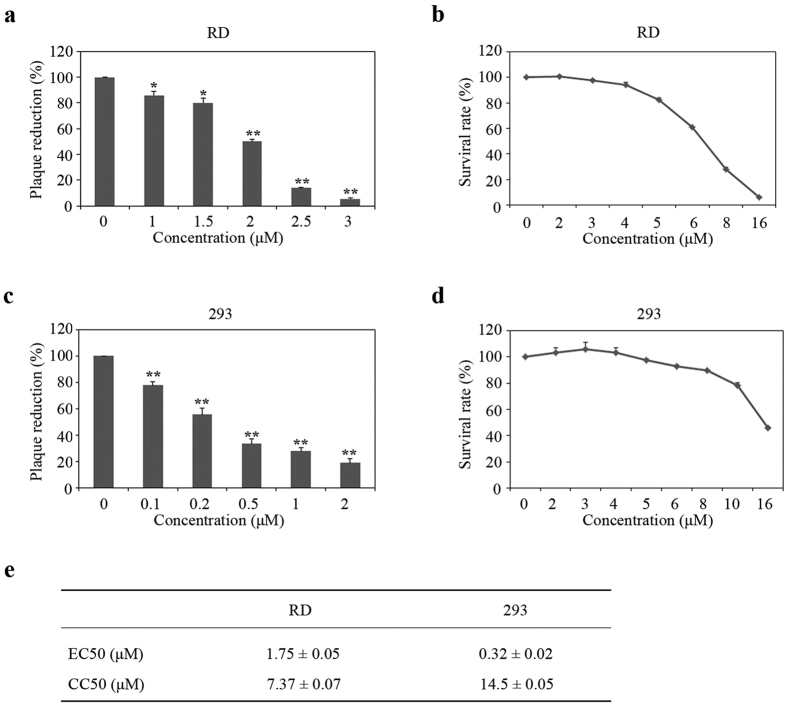
Amphotericin B inhibits EV71 production. (**a**) RD cells were pre-incubated with Amphotericin B at increasing concentrations for 2 h, and then infected with EV71 at an MOI of 0.05. After 24 h, culture medium was collected and viral titers were determined in plaque assays. The data represent 3 independent experiments, and error bars represent SD (*P < 0.05, **P < 0.01, *t* test). (**b**) Cytotoxicity of Amphotericin B on RD cells was measured by performing CellTiter-Glo^®^ Luminescent Cell Viability Assay. (**c**) Effect of Amphotericin B on the production of EV71 in 293 cells. The data represent 3 independent experiments, and error bars represent SD (**P < 0.01, *t* test). (**d**) Effects of Amphotericin B on the viability of 293 cells. (**e**) EC50 and IC50 of Amphotericin B on EV71 in RD and 293 cells.

We next measured the levels of EV71 production under Amphotericin B treatment. Titers of EV71 that was produced by RD cells or 293 cells were determined in the plaque assay. Figure 2a show that Amphotericin B drastically reduced the production of EV71 in RD cells with an EC50 (50% effective concentration) of 1.75 ± 0.05 μM. Similar trend of inhibition by Amphotericin B was observed in 293 cells with an EC50 of 0.32 ± 0.02 μM (Fig. 2c). To exclude the possibility that the inhibition of viral production is a result of Amphotericin B-mediated cytotoxicity, we measured cell viability under Amphotericin B treatment. The results showed that Amphotericin B had a CC50 (50% cytotoxic concentration) of 7.37 ± 0.07 μM in RD cells and 14.5 ± 0.05 μM in 293 cells, which are much higher than the EC50 values (Fig. 2b,d). These results demonstrate that Amphotericin B potently inhibits EV71 infection in both RD and 293 cells.

### Amphotericin B pretreatment does not affect the infectivity of EV71 virions

**Figure 3:**
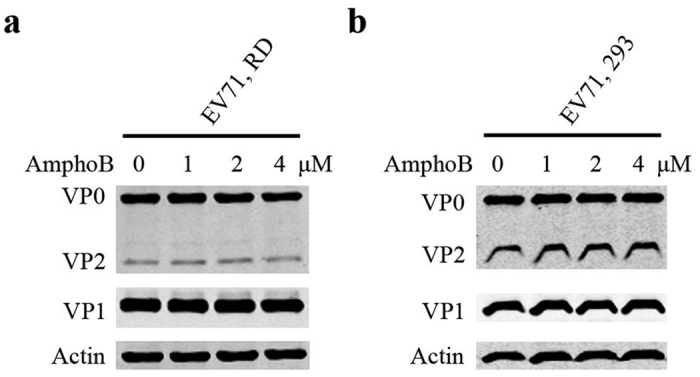
Effect of Amphotericin B on EV71 virions. EV71 virions were treated with increasing concentrations of Amphotericin B for 2 h prior to infecting RD cells at an MOI of 4 (**a**) or 293 cells at an MOI of 25 (**b**). The complete blots are presented in Supplementary Fig. 2.

It has been previously shown that pretreatment of enveloped viruses such as HIV-1 with Amphotericin B severely inhibited viral infection, whereas pretreatment of the target cells with Amphotericin B exerted minimal effect^43^. To test whether Amphotericin B inhibits HIV-1 and EV71 by a similar mechanism, we treated EV71 virions, but not the target cells, with different concentrations of Amphotericin B before applying EV71 to RD or 293 cells. In contrast to the profound inhibition of EV71 infection when target cells were pretreated with Amphotericin B as shown in Fig. 1, pretreatment of EV71 virions alone did not affect virus infection in RD (Fig. 3a) or 293 cells (Fig. 3b). These data suggest that pretreatment of EV71 virions did not affect structure of virions, and that Amphotericin B inhibits HIV-1 and EV71 by different mechanisms.

### Amphotericin B inhibits the early stage of EV71 infection

**Figure 4:**
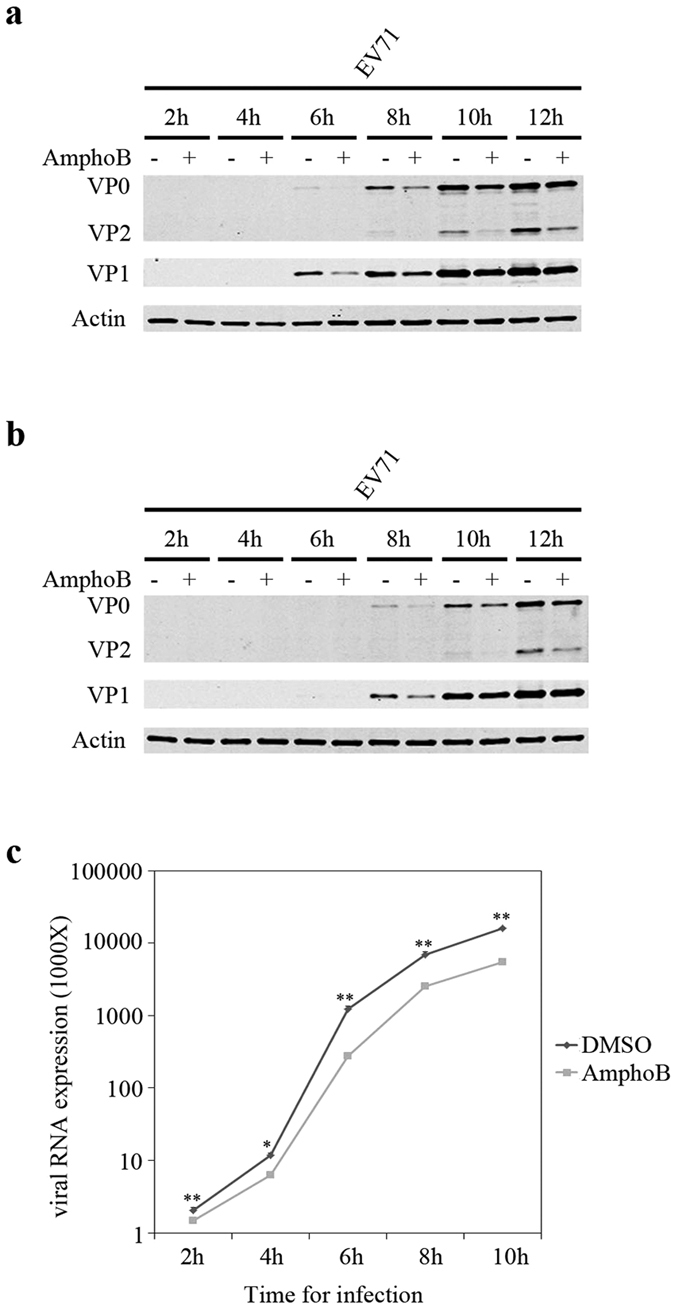
Amphotericin B inhibits the early stage of EV71 infection. (**a**) RD cells were infected with EV71 at an MOI of 4 in the absence or presence of 2 μM Amphotericin B. Levels of EV71 proteins in the infected cells were determined by Western blotting at 2, 4, 6, 8, 10, and 12 h post infection. (**b**) 293 cells were infected with EV71 at an MOI of 25 in the absence or presence of 1 μM Amphotericin B. Full-length blots are presented in Supplementary Fig. 3. (**c**) EV71 RNA in RD cells was quantified by qRT-PCR at 2, 4, 6, 8, 10 hpi. The data represented the copy number of EV71 RNA. Results shown are the average of three independent experiments. Error bars represent SD (*P < 0.05, **P < 0.01, *t* test).

Amphotericin B has been shown to inhibit different viruses at various stages of their life cycles^33^,^37^. We therefore examined which step of EV71 lifecycle was affected by Amphotericin B. RD cells or 293 cells were infected with EV71 in the absence or presence of Amphotericin B, and viral protein expression was measured by Western blotting. Expression of EV71 VP1 and VP2 was decreased at the earliest detectable time point (6 hpi in RD and 8 hpi in 293) (Fig. 4a,b). We next assessed the inhibition by quantifying the viral RNA through quantitative RT-PCR at 2, 4, 6, 8, and 10 h post infection (Fig. 4c). The results showed that the amount of EV71 RNA in the infected cells was reduced at 2 hpi under Amphotericin B treatment. These data suggest that Amphotericin B impedes an early step of EV71 life cycle.

### Amphotericin B prevents the binding and internalization of EV71 virions to target cells

**Figure 5:**
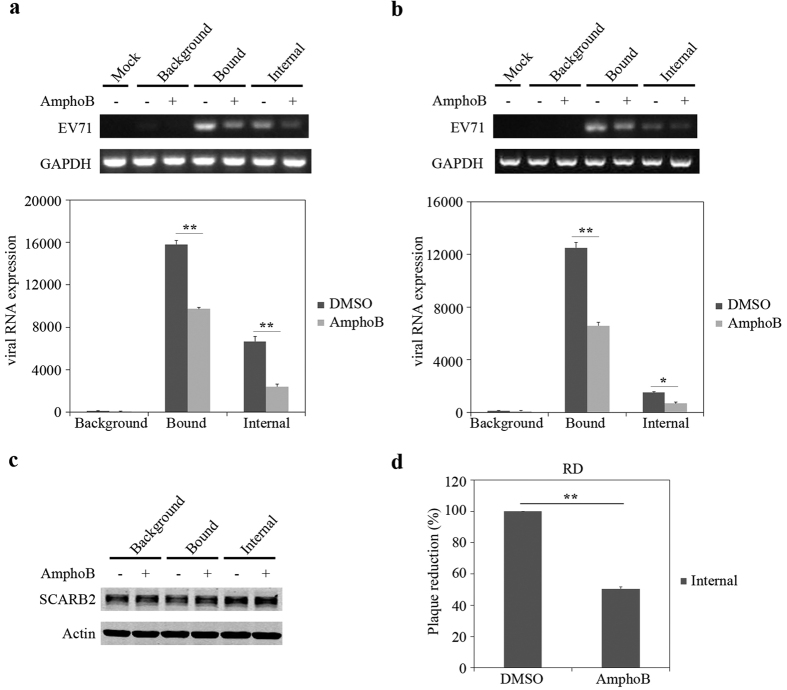
Amphotericin B impedes the binding and internalization of EV71 virions to host cells. EV71 was incubated with Amphotericin B pretreated RD (**a**) and 293 cells (**b**). Then background, bound, internal cells (described in materials and methods) were lysed for RNA extraction. Viral RNA was quantified by semi-RT PCR (upper panel) and viral RNA copy number was quantified by quantitative RT-PCR (lower panel). The results are plotted relative to virus background in DMSO treated cells. The data represent 3 independent experiments, and error bars represent SD (*P < 0.05, **P < 0.01, *t* test). (**c**) Levels of SCARB2 expression in background, bound, internal cells were determined by Western blotting. Full-length gels are presented in Supplementary Fig. 4. (**d**) The internalized viral titers were determined in plaque assays. The internal cells were collected at 10 hpi, and proceeded plaque assay after frozen and thaw. Cells treated with DMSO were set as 100%. The data represent 3 independent experiments, and error bars represent SD (**P < 0.01, *t* test).

To further identify which step of EV71 infection was inhibited by Amphotericin B, we examined binding of EV71 particles to target cells and viral internalization. Virus binding assay was performed by incubating EV71 viruses with RD or 293 cells with or without Amphotericin B at 4 °C as previously described^44^,^45^. After extensive washing to remove unbound viruses, amounts of viruses that were associated with target cells under Amphotericin B treatment decreased to less than 60% of that measured in the absence of Amphotericin B (Fig. 5, Bound). Protease treatment of the target cells decreased the PCR signal, supporting cell-bound nature of these viruses (Fig. 5, Background). To examine virus uptake, cells were first incubated with EV71 at 4 °C, then transferred to 37 °C for one hour to allow virus internalization. Cells were then treated with protease to remove viruses that bound to cell surface (Fig. 5, Internal). The results showed significant reduction of EV71 internalization by Amphotericin B. We also examined the expression of EV71 receptor SCARB2 by western blotting and found that SCARB2 expression was not affected by Amphotericin B (Fig. 5c). In addition, the internalized viruses were quantitated in plaque assay. As shown in Fig. 5d, the internalized viruses were reduced as a result of Amphotericin B treatment. Together, these data suggest that Amphotericin B impairs the binding and internalization of EV71 virus to host cells without affecting viral receptor expression.

### Amphotericin B inhibits EV68 infection

**Figure 6:**
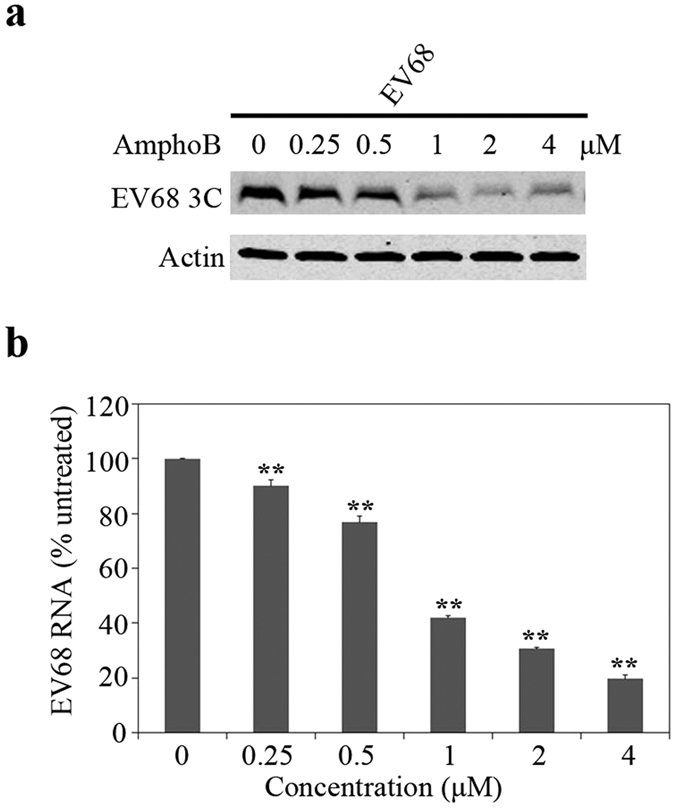
Amphotericin B inhibits EV68 infection. RD cells were pretreated with Amphotericin B at indicated concentrations for 2 h and then inoculated with EV68 at an MOI of 0.02. After 20 h, cells were harvested for western blotting and RNA extraction. (**a**) Levels of EV68 3C protein as determined by Western blotting at 20 hpi. Full-length blots are presented in Supplementary Fig. 5. (**b**) Levels of EV68 RNA were determined by qRT-PCR. Levels of viral RNA were normalized to GAPDH mRNA. Data represented percentage of Amphotericin B treatment to DMSO treatment. Results shown are the average of three independent experiments. Error bars represent SD (**P < 0.01, *t* test).

Over 1000 cases of severe respiratory diseases in pediatric patients were reported to associate with enterovirus 68 (EV68) infection in the fall of 2014^46^,^47^. We have therefore measured the effect of Amphotericin B on EV68 infection. RD cells were infected with EV68 in the absence or presence of Amphotericin B, and viral 3 C protein expression was examined by Western blotting. The results showed that expression of EV68 3 C protein was significantly diminished by Amphotericin B in a dose dependent manner (Fig. 6a). This inhibition was corroborated by the results of RT-PCR showing similar trend of decrease of viral RNA under the treatment of Amphotericin B (Fig. 6b). Together, these data demonstrate that Amphotericin B also inhibits EV68 infection.

## Discussion

A number of EV71 outbreaks have been reported since the first case of EV71 infection was documented in California in 1969^7^,^48^,^49^,^50^. Several recent EV71 outbreaks occurred in Asian countries and have resulted in substantial mortalities^8^,^9^,^10^,^11^,^12^. Effective antivirals to treat and control EV71 infection are still lacking, although efforts in this direction are under way^51^. In this study, we have demonstrated that Amphotericin B strongly inhibits EV71 infection. Amphotericin B showed an EC50 of 1.75 μM in RD cells and 0.32 μM in 293 cells, a CC50 of 7.37 μM in RD cells and 14.5 μM in 293 cells. In addition to EV71, Amphotericin B also potently inhibits EV68, a closely related enterovirus that has caused severe respiratory disease and is becoming a globally emerging pathogen in humans.

Amphotericin B inhibits HIV-1 and EV71 by different mechanisms. Different from HIV-1, pretreatment of EV71 virions with Amphotericin B did not affect EV71 infection. Upon binding to HIV-1 virion lipid bilayer, Amphotericin B had no effect on the levels of cholesterol, but may prevent the Env glycoprotein from undergoing conformational changes that are necessary to trigger membrane fusion. Alternatively, Amphotericin B-mediated inhibition could be due to direct binding of the compound to gp120 or to the ectodomain of gp41^43^. We did not observe any effect of Amphotericin B pretreatment on EV71 virions, likely because EV71 is a nonenveloped virus.

Given the prolonged process and the high costs to develop a new drug for clinical use, using the clinically approved drugs to treat EV71 infection is considered as an economical and efficient strategy. Amphotericin B is an antimicrobiotic agent, and has been used to treat many serious systemic fungal infections. Although Amphotericin B and its derivatives have been reported to inhibit VSV, HSV-1, Sindbis virus, vaccinia virus, HIV-1 and promote influenza virus replication, the effect of Amphotericin B on EV71 has not been reported. In this study, we observed strong inhibition of EV71 infection by Amphotericin B not only in RD but also in 293 cells. Amphotericin B also reduced infection of EV68. Results of virus binding assay suggested that Amphotericin B impaired the early phase of EV71 infection. This drug directly blocked the attachment and internalization of EV71 to host cells.

Side effects have been observed with high-dose amphotericin B treatment. Yet, these side effects are often transient and reasonably well tolerated in HIV-1 patients^52^. In the context of cancer chemotherapy, the benefits of amphotericin B treatment overweigh the adverse side effects. Several improvements have been made for Amphotericin B therapy. For example, liposomal formulation of Amphotericin B improved tolerability while maintaining treatment efficacy in patients suffering infections of fungi and HIV-1^53^. Importantly, Amphotericin B derivates such as MS-8209 (an N-methylglucamine salt of 1-deoxy-1-amino-4, 6-O-benzylidene-D-fructosyl Amphotericin B), AmBMU and AmBAU (the urea derivatives, Amphotericin B methyl urea and Amphotericin B amino urea) have much less toxicity (over 60 fold less) and deter the development of resistance^37^,^54^,^55^,^56^. These low-toxicity derivates hold the potential to treat young children with EV71 infection.

The primary goal of EV71 treatment is to prevent severe and fatal clinical outcomes. Our work supports the utility of Amphotericin B, an approved antibiotic, as a potential drug candidate to treat severe EV71 infections. Preclinical profiling is expected to determine the feasibility of clinical development of Amphotericin B as an EV71 therapeutic.

## Material and Methods

### Cell lines, viruses, drugs, and antibodies

Human muscular rhabdomyosarcoma (RD) cells and human embryonic kidney 293 cells were maintained in Dulbecco’s modified Eagle’s medium (DMEM) containing 10% FBS supplemented with L-glutamine, penicillin and streptomycin (Gibco BRL, Grand Island, NY, USA).

EV71 is a Fuyang strain (GenBank accession no. FJ439769.1). To conduct virus infections, cells were infected with EV71 at different MOIs (multiplicity of infection). Unbound viruses were washed off 2 h after infection. The enterovirus 68 (EV68) strain that was used in this study is a Beijing strain (GenBank accession no. KF726085). The reverse genetic system was utilized to produce influenza virus A/WSN/33 (H1N1)^57^.

Amphotericin B was purchased from Sigma–Aldrich. Mouse anti-VP1 antibody was purchased from Abnova, mouse anti-EV71 antibody from Millipore, anti-Actin antibody from Proteintech, anti-SCARB2 antibody from R&D systems, IRD Fluor 800-labeled IgG secondary antibodies from Li-Cor Inc., Lincoln, NE. The anti-EV68 3C antibody was obtained as previously described^58^.

### EV71 infection and inhibition assay in RD and 293 cells

RD or 293 cells were treated with DMSO or Amphotericin B of various concentrations for 2 h prior to EV71 infection at the indicated MOIs. Each infection was performed in triplicate. Cell viability was measured by performing CellTiter-Glo^®^ Luminescent Cell Viability Assay (Promega). Levels of viral proteins and viral RNA were determined by Western blotting and quantitative real-time PCR (qRT-PCR).

### Virus titration

Virus titers were measured in the plaque assay. Briefly, RD cells were seeded into 6-well plates. Infections were conducted with viruses of 10-fold serial dilutions (10^−1^ to 10^−8^). Infection was carried out for 2 h at 37 °C. The supernatants were then replaced with DMEM containing 1% agarose. 3 days after, the overlay medium was discarded. Cells were fixed in 4% paraformaldehyde followed by staining with Crystal violet. Viral plaques were scored by visual counting.

### Western blotting

Cells were first harvested and then lysed in RIPA buffer containing 150 mM NaCl, 25 mM Tris (pH 7.4), 1% NP-40, 0.25% sodium deoxycholate, 1 mM EDTA, 1 mM EGTA and a proteinase inhibitor cocktail (Roche). Proteins were separated on the 12% SDS-PAGE, and then transferred onto the nitrocellulose membrane (Millipore). After incubating in 5% milk, the membranes were probed with primary antibodies at 4 °C for overnight. The corresponding IRDye™ secondary antibodies were then applied to the membranes (Odyssey). After extensive washing, the membranes were scanned and analyzed using an Odyssey Infrared Imaging System (Li-Cor, Lincoln, NE).

### Quantitative real-time PCR

Total RNA were extracted from the EV71 infected cells using the RNAeasy Mini kit (Qiagen). Reverse transcriptions were then carried out using the Superscript First-Strand Synthesis System (Invitrogen). Viral RNA copy number was quantified by real-time PCR using Bio-Rad CFX96 touch q-PCR system. To generate a standard curve for cycle thresholds versus copy numbers, the pEGFP-VP1 plasmid was serially diluted to different concentrations. Primers for the amplification of VP1 gene were 5′-AGATAGGGTGGCAGATGTAATTGAAAG-3′ and 5′-TAGCATTTGATGATGCTCCAATTTCAG-3′.

### Virus binding assay

Virus binding was assessed as previously described with some modifications^44^,^45^. Briefly, RD cells were seeded in 6-well plates (8 × 10^5^ cells/well). The next day, cells were first treated with DMSO or Amphotericin B for 2 h, and then washed with cold PBS, followed by incubation with 1 ml of binding buffer (PBS containing 1% BSA and 0.1% sodium azide) for 10 min on ice. Cells were then incubated with viruses at the indicated MOI for 1 h on ice. One group of cells were washed with PBS to remove unbound viruses and used to determine levels of bound virions (Bound). The second group of cells were treated with trypsin for 3 minutes to remove bound virions and the results serve as the background of the assay (Background). The third group of cells were cultured at 37 °C for one hour to allow virus internalization before treated with trypsin to remove any virions that still bound to cell surface (Internal). Total RNA was then extracted and the levels of viral RNA were determined by quantitative RT-PCR. Three independent virus binding experiments were performed for each condition.

## Additional Information

**How to cite this article**: Xu, F. *et al*. Amphotericin B Inhibits Enterovirus 71 Replication by Impeding Viral Entry. *Sci. Rep.* **6**, 33150; doi: 10.1038/srep33150 (2016).

## Supplementary Material

Supplementary Information
