# Supplementary material for: Amphotericin B Inhibits Enterovirus 71 Replication by Impeding Viral Entry
Source: Sci Rep. 2016 Sep 9;6:33150. doi: 10.1038/srep33150 (PMC5016833; doi:10.1038/srep33150)

**Supplementary Information**

**Amphotericin B Inhibits Enterovirus 71 Replication by Impeding Viral Entry**

Fengwen Xu^1,#^, Xiaoxiao Zhao^1,#^, Siqi Hu^1^, Jian Li^1^, Lijuan Yin^1^, Shan Mei^1^, Tingting Liu^1^, Ying Wang^1^, Lili Ren^1^, Shan Cen^2^, Zhendong Zhao^1^, Jianwei Wang^1^, Qi Jin^1^, Chen Liang^1,3^, Bin Ai^4,*^ and Fei Guo^1,*^

^1^ MOH Key Laboratory of Systems Biology of Pathogens，Institute of Pathogen Biology, and Center for AIDS Research, Chinese Academy of Medical Sciences & Peking Union Medical College, Beijing, P. R. China.

^2^ Institute of Medicinal Biotechnology, Chinese Academy of Medical Sciences & Peking Union Medical College, Beijing, P. R. China.

^3^ Lady Davis Institute, Jewish General Hospital, Montreal, Qc, Canada H3T 1E2.

^4^ Department of Medical Oncology, Beijing Hospital, Beijing, P. R. China.

^# *^ These authors contributed equally to this work. Correspondence and requests for materials should be addressed to B.A. (email: docaibin@163.com) or F.G. (email: guofei@ipb.pumc.edu.cn)


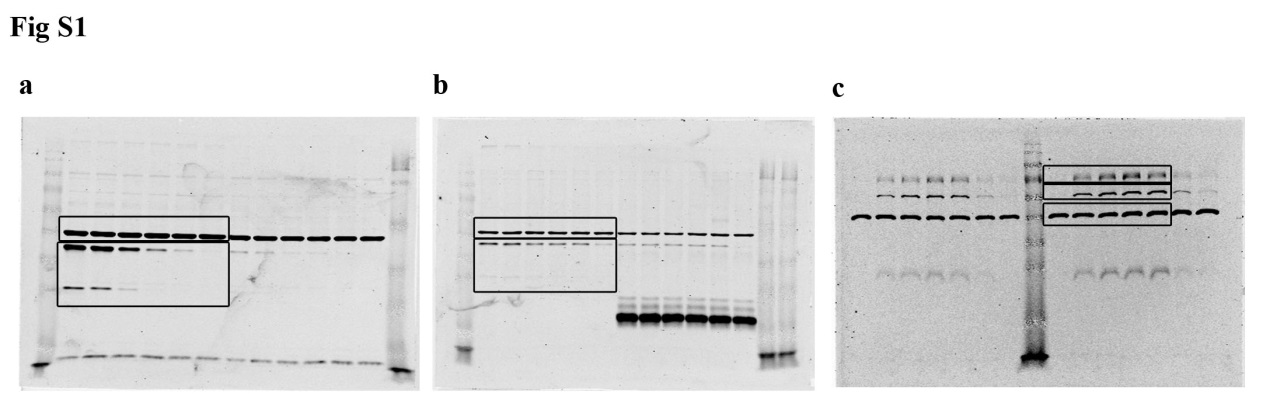


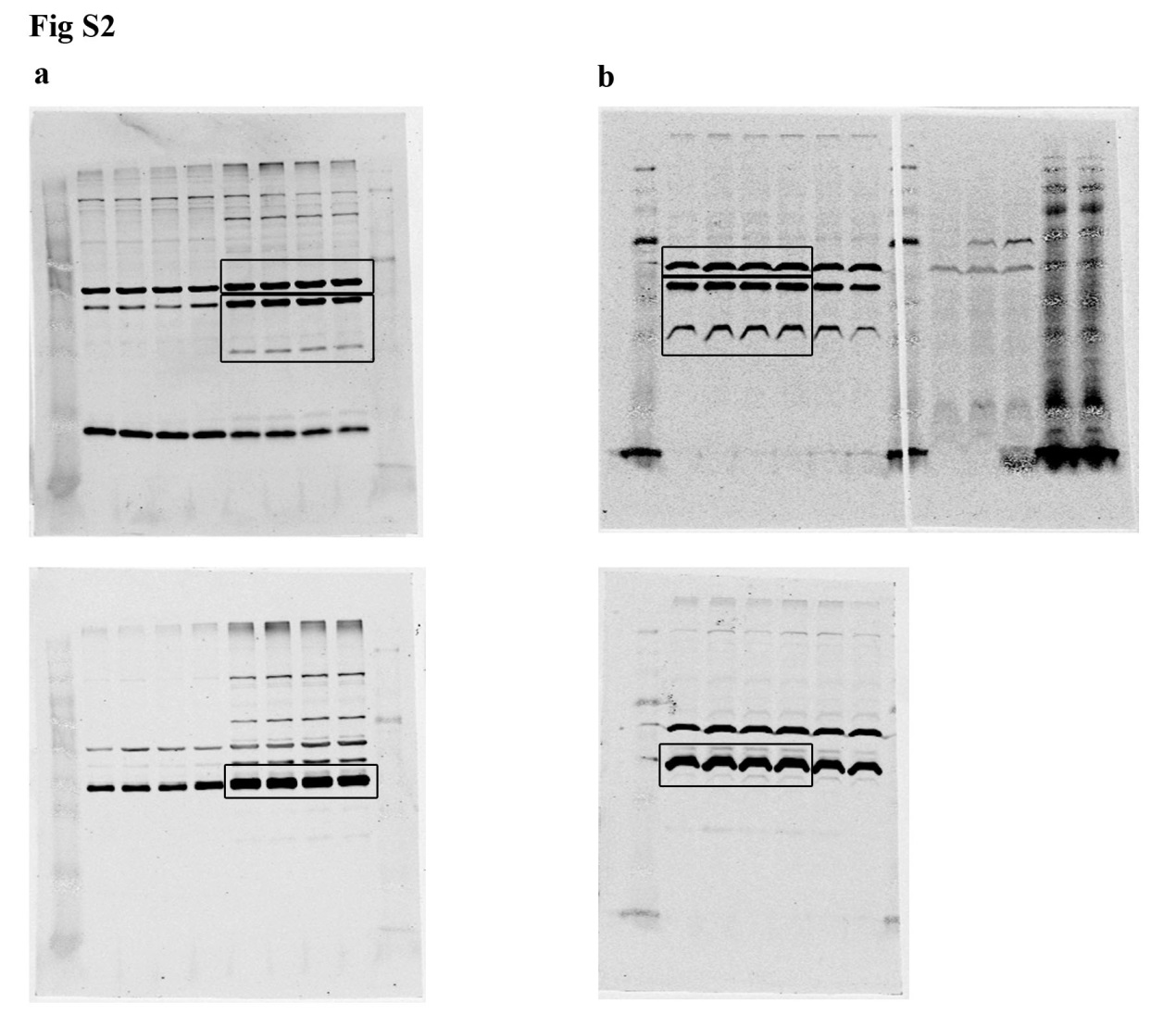


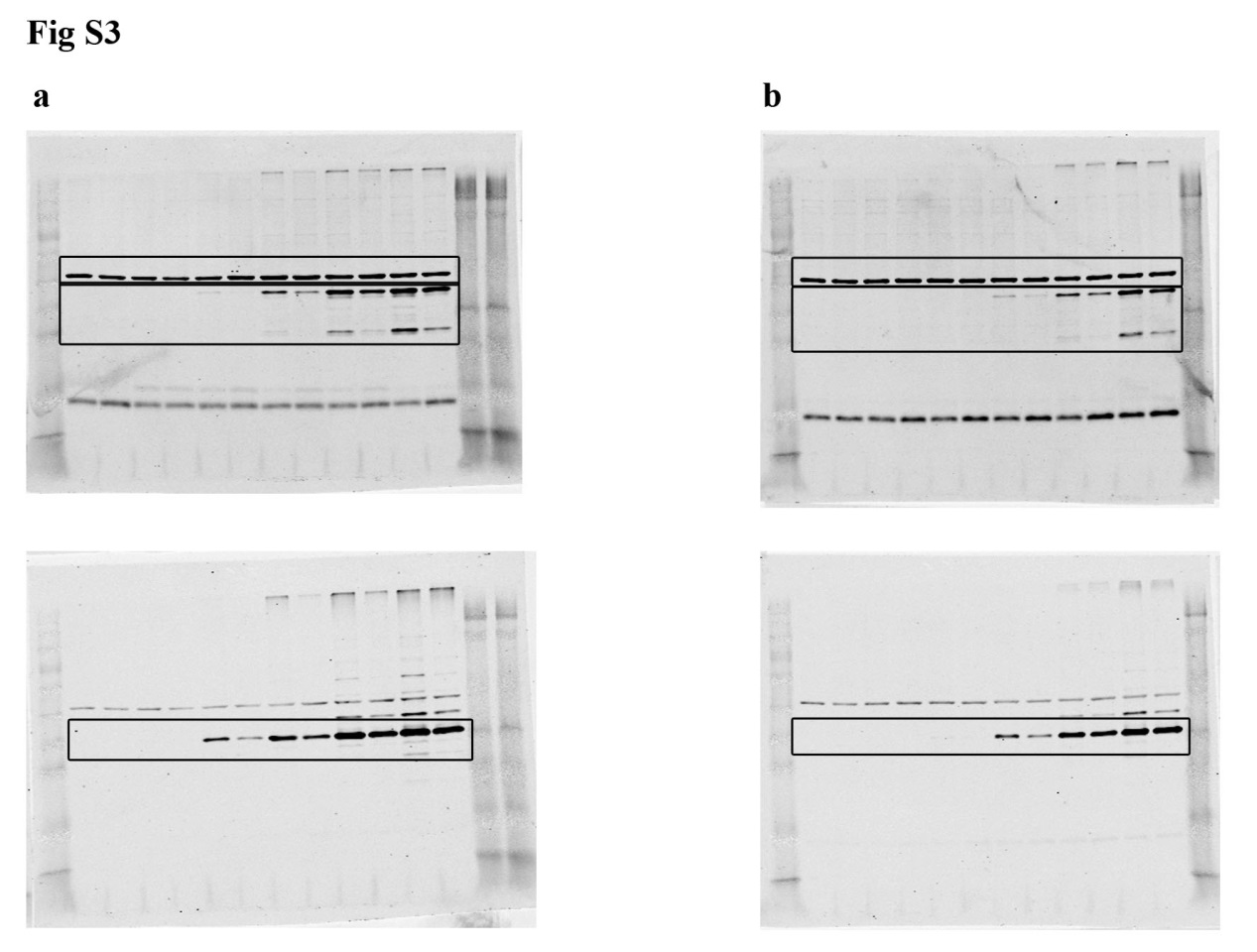


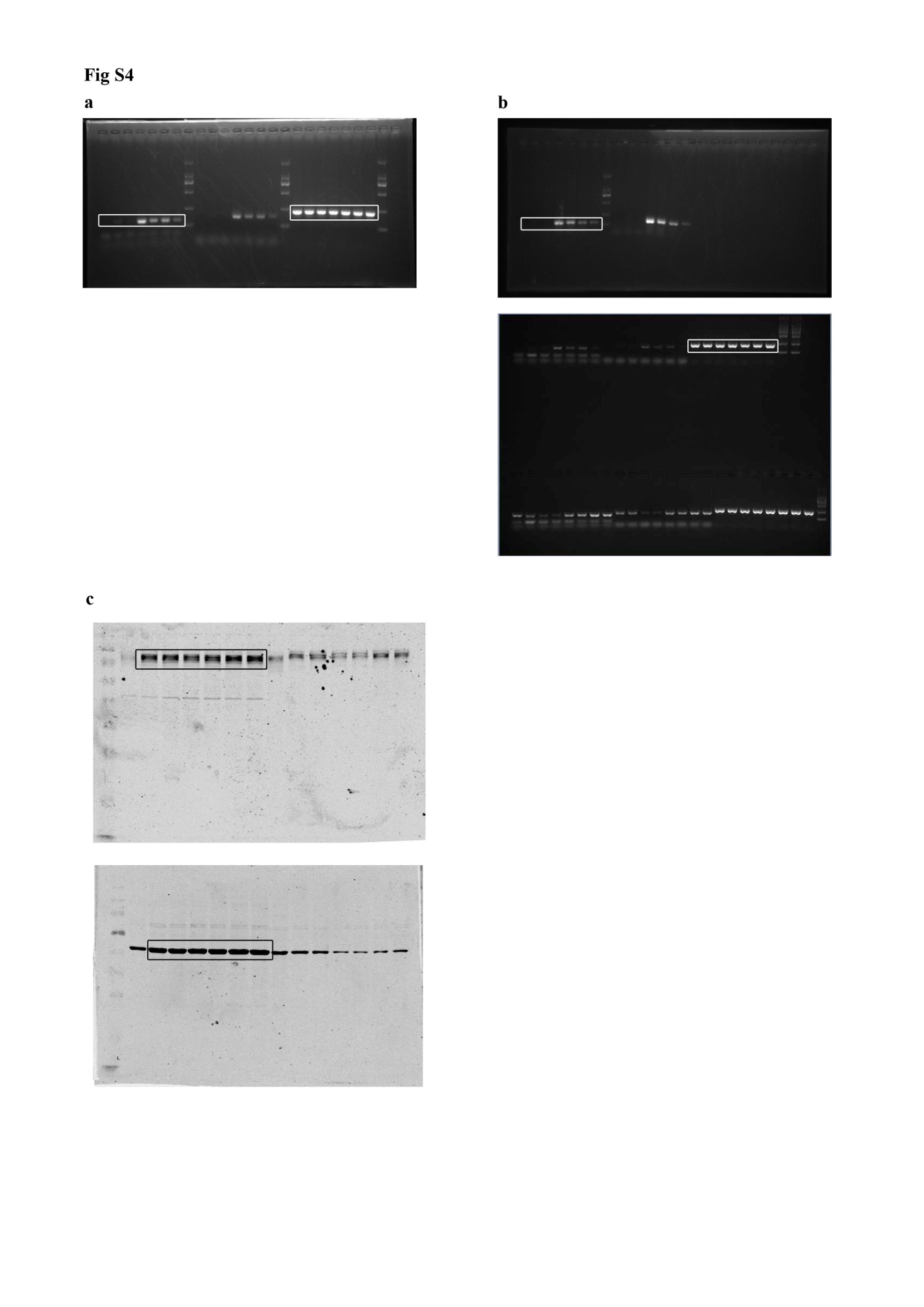


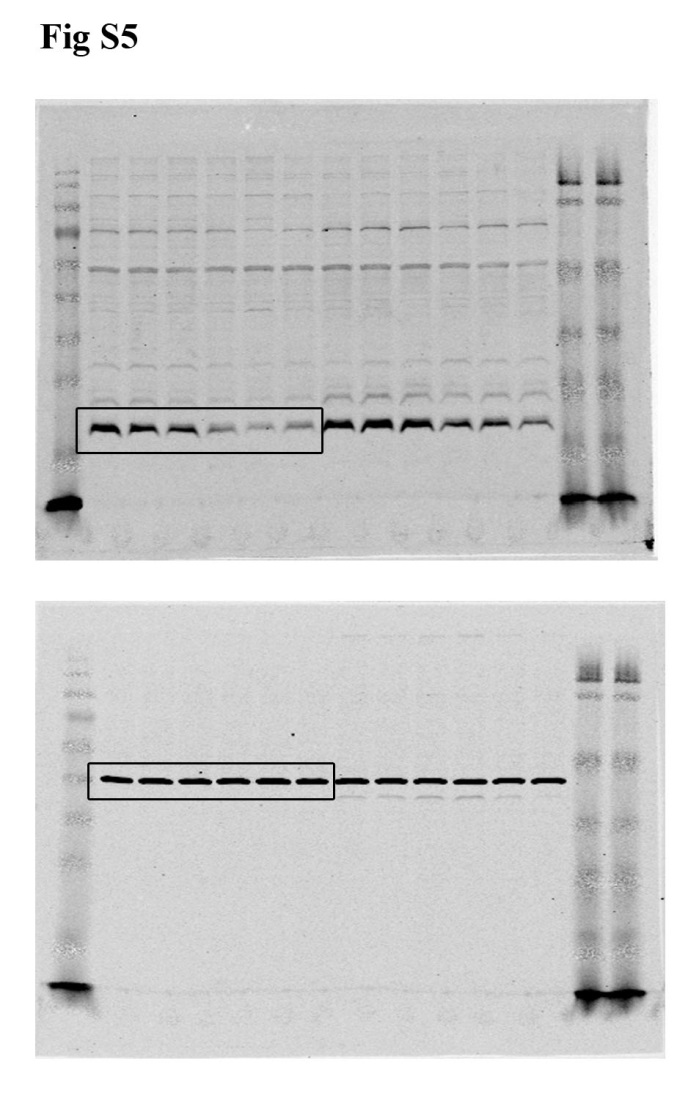

Supplement: Supplementary Information [file srep33150-s1.doc]
